# Claudin-3 inhibits tumor-induced lymphangiogenesis via regulating the PI3K signaling pathway in lymphatic endothelial cells

**DOI:** 10.1038/s41598-022-22156-6

**Published:** 2022-10-19

**Authors:** Ningjing Lei, Yanru Cheng, Jiajia Wan, Rosel Blasig, Anqi Li, Yueyue Bai, Reiner F. Haseloff, Ingolf E. Blasig, Linyu Zhu, Zhihai Qin

**Affiliations:** 1 Medical Research Center, The First Affiliated Hospital of Zhengzhou University, Zhengzhou University, No. 1 Jianshe Road, Zhengzhou, 450052 Henan China; 2 School of Basic Medical Sciences, Zhengzhou University, No. 100 Kexue Road, Zhengzhou, 450001 China; 3 Leibniz-Forschungsinstitut Für Molekulare Pharmakologie (FMP), Robert-Rössle-Straße 10, 13125 Berlin, Germany

**Keywords:** Cancer microenvironment, Metastasis

## Abstract

Claudin-3 is a tight junction protein that has often been associated with the progression and metastasis of various tumors. Here, the role of claudin-3 in tumor-induced lymphangiogenesis is investigated. We found an increased lymphangiogenesis in the B16F10 tumor in claudin-3 knockout mice, accompanied by augmented melanoma cell metastasis into sentinel lymph nodes. In vitro, the overexpression of claudin-3 on lymphatic endothelial cells inhibited tube formation by suppressing cell migration, resulting in restricted lymphangiogenesis. Further experiments showed that claudin-3 inhibited lymphatic endothelial cell migration by regulating the PI3K signaling pathway. Interestingly, the expression of claudin-3 in lymphatic endothelial cells is down-regulated by vascular endothelial growth factor C that is often present in the tumor microenvironment. This study indicates that claudin-3 plays an important role as a signaling molecule in lymphatic endothelial cell activity associated with tumor lymphangiogenesis, which may further contribute to melanoma metastasis.

## Introduction

The vasculature system in the tumor microenvironment contributes to the tumor progression by inducing vessel growth. In many types of tumor, such as melanoma, lymphangiogenesis is an important aspect that provide routes for tumor lymphatic metastasis^1^. More and more studies have indicated that melanoma lymphangiogenesis is closely related to tumor lymph node metastasis, which is critical for further diagnosis and prognosis^2–4^.

The mechanisms of tumor lymphangiogenesis seem to be very complex^5,6^, and lymphatic endothelial cells (LECs) proliferation, migration and tube formation are key events related to newly formed vessels^7,8^. For instance, activation of the PI3K signaling pathway leads to the phosphorylation of the downstream substrates like AKT and ERK, which can promote LECs migration that eventually contributing to lymphangiogenesis^9–11^. In addition, vascular endothelial growth factor C (VEGF-C) and its receptor VEGFR-3 also promote tumor lymphangiogenesis by activating the related signaling pathways^12,13^.

Tight junction (TJ) proteins play important roles in the maintenance of cell activities and vascular integrity^14^. Different TJ proteins are reported and investigated on endothelial cells. For instance, TJ component junctional adhesion molecule-B (JAM-B) expresses in post capillary endothelial cells and lymphatic vessels^15^. Another component occludin on endothelial cells correlates with the permeability function^16^. The claudin family belongs to TJ proteins, and several members such as claudin-5 and claudin-14 are found to be expressed on endothelial cells with multiple functions^17,18^. It is also reported that diminished TJ integrity occurs in endothelial cells to allow intravasation and extravasation^19,20^.

Claudin-3 is a member of the claudin family expressed on epithelial cells and endothelial^17,21^. Most studies focus on the expression of claudin-3 on tumor epithelial cells affect tumor metastasis. For instance, claudin-3 inhibits lung squamous cancer cell migration by suppressing the Wnt/β-catenin signaling pathway^22^. Others also study the association of claudin-3 and tumor progression without distinguishing the expression of the protein on tumor cells or stromal cells^23,24^. In addition, claudin-3 expressed on endothelial cells has been investigated in many studies, especially the regulation of the blood–brain barrier (BBB)^25,26^. Thus, functional assessment of tumor cell extrinsic roles for claudin-3 in tumor metastasis is unclear.

In the present study, we built a B16F10 metastasis tumor model using claudin-3-null (claudin-3^−/−)^ mice. We found that the knockout of claudin-3 in mice increased B16F10 melanoma cell metastasized into the draining lymph nodes significantly compared to control claudin-3^+/+^ mice. We then detected lymphatic vessels in tumor site and found increased lymphangiogenesis in claudin-3^−/−^ mice compared with the control. Immunohistochemistry (IHC) results indicated that claudin-3 was expressed on lymphatic vessels in B16F10 tumor tissues. And we also detected the expression of claudin-3 protein on the LECs. Functions and mechanisms associated with lymphangiogenesis of claudin-3 on LECs were explored. Our results indicated that loss of claudin-3 in the tumor microenvironment increased melanoma lymphatic metastasis and upregulated tumor lymphangiogenesis. These novel functions of claudin-3 on LECs are worth further analysis that may provide a new target for restricting melanoma metastasis.

## Materials and methods

### Animal studies

Wild type control mice claudin-3^+/+^ and claudin-3 knock out (claudin-3^−/−^) mice on the C57BL/6 background have been described previously^27,28^. Mice samples were analyzed by nucleic acid electrophoresis gel to confirm the homozygous background (Supplementary Fig. 1a). In addition, loss of claudin-3 protein in the null mice was examined on multiple tissue samples by immunohistochemistry staining (Supplementary Fig. 1b and c). All mice were bred in a specific pathogen-free environment at the First Affiliated Hospital of Zhengzhou University (Zhengzhou, China). Sex and age matched mice (female, 6 to 8 weeks old) were used. Animal experiments were carried out with the approval of the Institutional Laboratory Animal Care and Use Committee of the First Affiliated Hospital of Zhengzhou University (No. 2021-KY-1088-002). All methods in this study were carried out in accordance with the guidelines of the Institutional Laboratory Animal Care and Use Committee of the First Affiliated Hospital of Zhengzhou University, and in compliance with the ARRIVE guidelines.

### Cell line

The melanoma cell line B16F10 was purchased from ATCC (Manassas, VA, USA) and cultured in RPMI 1640 medium (Gibco, MA, USA) with 10% fetal calf serum (PAA, Bentley, Australia) at 37 °C with 5% CO_2_. The B16F10 cells were transfected with mCherry plasmid to show detectable fluorescence (mCherry-B16F10). The lymphatic endothelial cell line SVEC4-10 was kindly provided by Dr. Mingzhao Zhu (Institute of Biophysics, Chinese Academy of Sciences) and cultured in DMEM medium (Gibco, MA, USA) supplemented with 10% fetal bovine serum (FBS) (PAA, Bentley, Australia) at 37 °C with 5% CO_2_.

### Reagents

The PI3K inhibitor LY294002 (cat. #HY-10108) and the ERK inhibitor PD98059 (cat. #HY-12028) was purchased from MedChemExpress (NJ, USA). Recombinant VEGF-C (cat. #100-20CD) was purchased from Peprotech (NJ, USA).

### Tumor transplantation model

5 × 10^5^ mCherry-B16F10 in single-cell solution was mixed with matrigel (Corning, NY, US) (1:1) injected subcutaneously into footpad of mice (n = 5 mice per group, repeated three times). Tumor growth was monitored every 2 days after day 11. Tumor volumes were calculated as length × width^2^ × 0.5. Mice were sacrificed at day 28 when the tumor reached about 800 mm^3^. The primary tumor, popliteal lymph node (LN), inguinal LN, spleen, liver, and lung tissues were enucleated. Popliteal LNs were imaged with IVIS Spectrum Imaging System (PerkinElmer, MA, USA) to evaluate lymphatic metastasis. The primary tumor tissues were embedded in Tissue Tek O.C.T (SAKURA, CA, USA, cat. #4583) for IHC sample preparation.

### Immunohistochemistry (IHC) staining

Lymph nodes and tumor tissues from the transplantation model were prepared for frozen sections as described previously^29^. For immunofluorescence staining, rabbit anti-LYVE1 (Abcam, MA, USA, cat. #ab14917), rat anti-CD31 (BD, NJ, USA, cat. #550274) and rabbit anti-claudin-3 (Abcam, MA, USA, cat. #ab15102) antibodies were used as primary antibodies. The secondary antibodies were Alexa FluorTM488 donkey anti-rabbit-IgG (Invitrogen, CA, USA, cat. #A21206) and Alexa Fluor TM488 donkey anti-rat IgG (Invitrogen, CA, USA, cat. #A21208), respectively. For quantitative analysis of vessel density, 15–18 optical fields were taken for each tumor sections (200 × magnification) using the Vectra machine (Perkin Elmer, Waltham, MA, USA), with 3 tumors in each group selected randomly. The lymphatic vessel area was calculated by staining the tumor sections for LYVE1. The percentage of LYVE1 + staining area per field was quantified using Image J and analyzed by the GraphPad software.

### Transfection of claudin-3 overexpression in SVEC4-10 cell

SVEC4-10 cells stably overexpress claudin-3 by lentivirus infection. HEK293T cells were transfected with pCDH-EF1-MCS-T2A-copGFP or pCDH-EF1-MCS-T2A-copGFP-CLND3, enveloped plasmid pMD2.G and packaging plasmid psPAX2. The supernatants containing lentivirus of HEK293T were harvested at 48 h post transfection. Supernatants were collected and filtered with 0.45 μm binding filter (Millipore, MA, USA), then mixed with culture medium (1:1) and added into cells plated in monolayer in a 6-well plate with 50–60% confluence. After infection, cells were placed in an incubator for 24 h. Discard the original medium and replace it with fresh culture medium. The culture supernatant was collected after 48 h. GFP positive cells were selected by fluorescence-activated cell sorting by FACSAria III (BD Biosaciences, USA).

### Knockdown of claudin-3 by lentivirus encoding claudin-3 short hairpin RNA in SVEC4-10 cell

Two target sequences to knockdown claudin-3 were obtained from Genechem (Shanghai, China). The plasmids were transfected into SVEC4-10 cells using the Lipofectamine 3000 transfection kit (Thermo Scientific, MA, USA) according to the manufacturer’s instructions. The sequences for claudin-3 shRNA are as followed: 5′-CCA ACU GCG UACA AGA CGA-3′ (shRNA1) and 5′-CCC ACC AAG AUC CUC UAU UTT-3′ (shRNA2).

### Quantitative real-time polymerase chain reaction (PCR)

Total RNA was extracted from the primary tumor tissues of the above model by adding Trizol reagent (Life Technologies, MD, USA) according to the manufacturer’s instructions. cDNA was synthesized by reverse transcription using PrimeScriptTM RT Master Mix (Applied TaKaRa, Otsu, Shiga, Japan) according to the manufacturer’s instructions. Quantitative real-time PCR was performed using SYBR Green Master Mix (Applied TaKaRa, Otsu, Shiga, Japan) and ABI PRISM 7300HT Sequence Detection System (Applied Biosystems, Foster City, CA, USA). Designed primers for Claudin-3: primer 1, 5′-GCA CCC ACC AAG ATC CTC TA-3′ and TCG TCT GTC ACC ATC TGG AA-3′; primer 2, 5′-CGT ACA AGA CGA GAC GGC CAA G-3′ and CAC GTA CAA CCC AGC TCC CAT C-3′; primers for GAPDH: 5′-CAT CAA GAA GGT GGT GAA GC-3′ and 5′-CCT GTT GCT GTA GCC GTA TT-3′.

### Western blot analysis

Cells were lysed by RIPA solution and protein samples (20–40 μg) were electrophoresed on 10% or 12% sodium dodecyl sulfate–polyacrylamide gels. The proteins were then transferred onto nitrocellulose membrane (GE Healthcare, MA, USA). Membrane blots were pre-stained with fast green and cut at desired positions according to the molecular sizes. Cut blots were incubated with primary antibodies including anti-claudin-3 (1: 1 000, Abcam, MA, USA, cat. #ab15102), β-actin (1: 8 000, Sigma-Aldrich, MO, USA, cat. #A5441), phosphor-AKT (1: 1 000, Cell Signaling Technology, MA, USA, cat. #: 4060), phosphor-ERK (1: 1 000, Cell Signaling Technology, CAT#: 4377, MA, USA), AKT (1: 1 000, Cell Signaling Technology, MA, USA, cat. #9272), ERK (1: 1 000, Cell Signaling Technology, MA, USA, cat. #4695), at 4 Celsius degree overnight. Anti-mouse or anti-rabbit secondary antibodies conjugated to horseradish peroxidase (IgG, 1:10 000) were used to bind for 60 min at room temperature. Immunoreactive proteins were visualized with an enhanced chemilluminescence visualization kit (Thermo Scientific, MA, USA). All samples were performed with 3–4 replicates.

### Proliferation assay

The cell proliferation was determined using the Cell Counting Kit-8 (Sigma-Aldrich, St Gallen, Switzerland) according to the manufacturers’ instructions. In brief, cells were plated in a 96-well plate with the density of 2 × 10^4^ cells/well, and cultured for 48 or 72 h before the addition of CCK-8 solution. Each group included 5 replicates and repeated 3 independent times. To each well, 10 μL CCK-8 solution was added and cells were cultured for additional 4 h. The absorbance of the water-soluble formazan product was measured at a wavelength of 450 nm using a microplate reader (Bio-rad, CA, USA).

### Transwell assay

The cells after transfection were re-suspended with the cell density adjusted to 2 × 10^4^ cells/mL. Next, the cell suspension (100 μL) was added to the upper chamber of the Transwell chamber with DMEM medium containing 1% FBS, while 600 μL DMEM medium containing 10% FBS was added to the lower chamber for culture in a 37 °C, 5% CO_2_ incubator for 24 h. Then the Transwell chamber was taken out, cells on the inside of upper chamber were wiped with a cotton swab, and the remaining cells were fixed with 4% paraformaldehyde for 15 min, stained with 0.5% crystal violet solution (made with methanol) for 15 min and washed 3 times with PBS. A total of 5 fields were randomly selected for photographing under the inverted microscope (LEICA, Wetzlar, Germany). The trans-membrane cells were counted with 3 replicates in each group and the experiment was repeated 3 times.

### Wound healing assay

Cells after transfection were collected, and the cell suspension was inoculated into a 96-well plate at a cell density of 1 × 10^5^ cells/well. Each group included 5 replicates and repeated 3 independent times. When the cell confluence reached 100%, the scratch wounds were performed with IncuCyte (Sartorius, Germany) wound maker. Cells were washed twice with 1 × PBS to remove the damaged cells, followed by adding DMEM with 10% FBS or other treatments. The scratch wounds were photographed by IncuCyte live-cell imaging system every 4 h. The wound widths were analyzed using IncuCyte analysis software.

### Tube formation assay

Matrigel (BD Biosciences, CA, USA, cat. #356234) and serum-free DMEM were mixed at a ratio 1:1. A 60 uL volume of the mixture was pipetted in a 96-well plate and polymerized at 37 °C for 30 min. SVEC4-10 cells in 100 uL of medium were added to each well. Each group included 3 replicates and repeated 3 independent times. The quantitative analysis of total length and the number of master junctions were measured using Image J (Media Cybernetics, MD, USA) software package.

### Statistical analysis

Statistical analysis was performed by GraphPad Prism 9 software (https://www.graphpad.com/scientific-software/prism). The two-tailed Student’s t test was used to compare the statistical significance between two groups. The one-way analysis of variance (ANOVA) was used to analyze the differences of three groups and more than three groups of data. The measured data are expressed in the form of mean ± SEM. Statistically significant differences are indicated as follows: *p < 0.05, **p < 0.01, ***p < 0.001.

## Results

### Claudin-3 deficiency augments lymphatic metastasis of B16F10 melanoma cells

**Figure 1:**
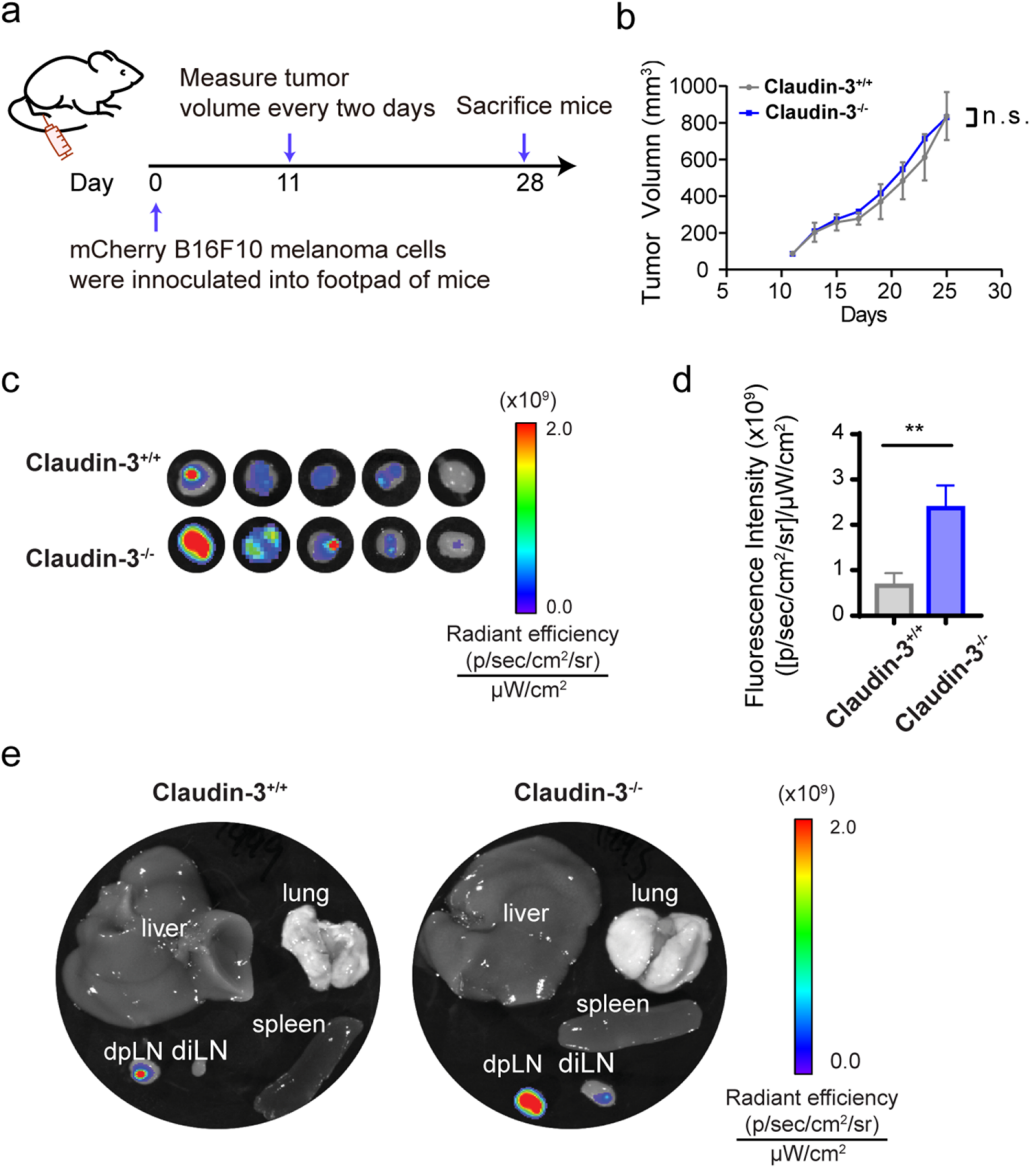
Loss of claudin-3 augmented B16F10 melanoma lymphatic metastasis. (**a**) Schematic flowchart shows the establishment of B16F10 melanoma transplantation model in mice. (**b**) The tumor volume was measured and calculated after day 9 as described in the method part. (ns: no significance) (n = 5 mice per group) (**c**) mCherry fluorescence was detected by IVIS in ipsilateral popliteal lymph nodes (dpLN) of the footpad injection site from claudin-3^−/−^ and claudin-3^+/+^ mice. (n = 5 mice per group) (**d**) Quantification of metastasized mCherry labeled B16F10 cells in dpLN was measured by IVIS. Statistical analysis of fluorescence was conducted. Values are shown as the mean ± SEM. (**p < 0.01) (n = 5 mice per group) (**e**) After sacrificing the mice, ipsilateral popliteal lymph node (dpLN), ipsilateral inguinal lymph node (diLN), liver, lung, and spleen were collected. Representative images show the fluorescence examined by IVIS.

To study the association between claudin-3 and melanoma metastasis, we used claudin-3^−/−^ mice and control claudin-3^+/+^ mice to establish a popliteal lymph node metastasis model by injecting 5 × 10^5^ mCherry-labeled melanoma cell line B16F10 subcutaneously into the footpad (Fig. 1a). The primary tumor volume between claudin-3^−/−^ and claudin-3^+/+^ mice showed no statistical difference (Fig. 1b). We then examined the metastasis of mCherry-B16F10 cells in lymph nodes, liver, lung and spleen tissues after sacrificing the mice. Increased tumor metastasis could be observed in the ipsilateral draining popliteal lymph node (dpLN) (Fig. 1c). Metastasized tumor cells in dpLN were quantified by the fluorescence (Fig. 1d). Ex vivo images indicated that besides dpLN, fluorescence intensity also increased in ipsilateral inguinal lymph nodes (diLN) of claudin-3^−/−^ mice compared to claudin-3^+/+^ mice, but no distant metastasis was detected in other organs (Fig. 1e). The incidences of metastasis in both dpLNs and diLNs were analyzed (Supplementary Fig. 2). Thus, the results showed that loss of claudin-3 could increase lymphatic metastasis of B16F10 cells into the regional lymph nodes.

### Claudin-3 deficiency leads to increased tumor lymphangiogenesis in mice

**Figure 2:**
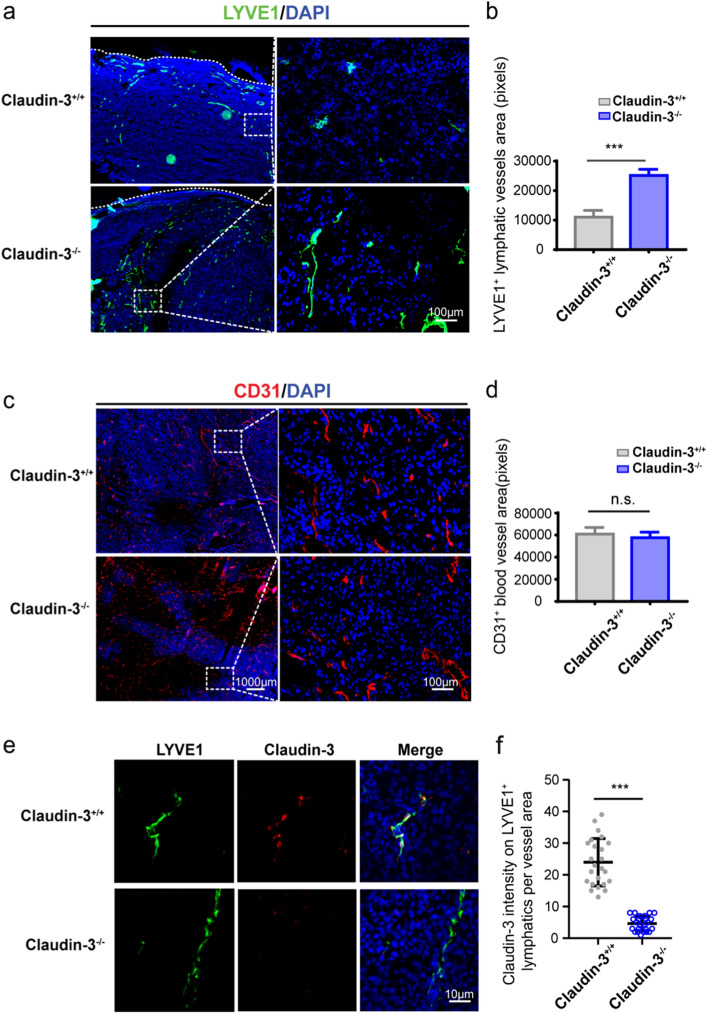
Loss of claudin-3 promoted lymphangiogenesis in primary tumor tissue. (**a**) Representative images of the lymphatic vessel marker LYVE-1 staining (green staining) in melanoma tissue of claudin-3^+/+^ and claudin-3^−/−^ mice. Dashed lines indicate margin of tumor tissue. Left panel: 4X picture; scale bar, 1000 μm. Right panel: 20X picture; scale bar, 100 μm. (**b**) Statistical analysis of the lymphatic vessels under a high magnification (20X) fluorescence microscope. Values are shown as the mean ± SEM. (***p < 0.001) (n = 20 fields per group) (**c**) Representative images of CD31 staining (red staining) in melanoma tissue of claudin-3^+/+^ and claudin-3^−/−^ mice. Left panel: 4X picture; scale bar, 1000 μm. Right panel: 20X picture; scale bar, 100 μm. (**d**) Statistical analysis of the blood vessels under a high magnification (20X) fluorescence microscope**.** Values are shown as the mean ± SEM. (ns: no significance) (n = 20 fields per group) **(e)** Co-staining of tumor tissue from the claudin-3^+/+^ and claudin-3^−/−^ mice with anti-LYVE-1 antibody (green staining) and anti-claudin-3 antibody (red staining). Scale bar, 10 μm. (**f**) Quantification of claudin-3 fluorescence intensity on LYVE-1 + lymphatics in **e.** (***p < 0.001) (n = 24 vessels per group).

Previous studies have shown that lymphatic vessels provide important routes in tumor metastasis^1,4^. To verify the routes for melanoma metastasis in our model, frozen sections of primary tumor were prepared and stained with lymphatic vessel marker anti-LYVE-1 and blood vessel marker anti-CD31, respectively. Vessels could be seen inside tumor tissues as well as in the adjacent tissues. More LYVE1^+^ area could be observed in claudin-3^−/−^ mice compared to the control mice, while CD31^+^ areas didn’t show an obvious difference (Fig. 2a,c). Quantification and statistical analysis by Inform software confirmed that the LYVE1^+^ area was increased in tumor sites of claudin-3^−/−^ mice indicating the presence of more lymphatic vessels existed (Fig. 2b). In contrast, no significant difference in the number of blood vessels was observed between claudin-3^−/−^ mice and control mice in tumor sites (Fig. 2d). The result showed that loss of claudin-3 led to increased lymphangiogenesis at primary tumor sites. To explore whether claudin-3 exerts a direct effect on lymphatic vessels, we first examined whether claudin-3 protein was expressed on lymphatic vessels. By immunofluorescence staining of the tumor tissues, we could see co-expression areas of LYVE-1 positive and claudin-3 positive staining in claudin-3^+/+^ mice (Fig. 2e,f). In addition, the co-localization of LYVE-1 positive and claudin-3 positive areas were also confirmed in normal skin tissues of wild type control mice (Supplementary Fig. 3). Thus, we focused on how claudin-3 affected lymphangiogenesis by examining its roles on LECs.

### Knockdown of claudin-3 in endothelial cells promotes lymphatic tube formation in vitro

**Figure 3:**
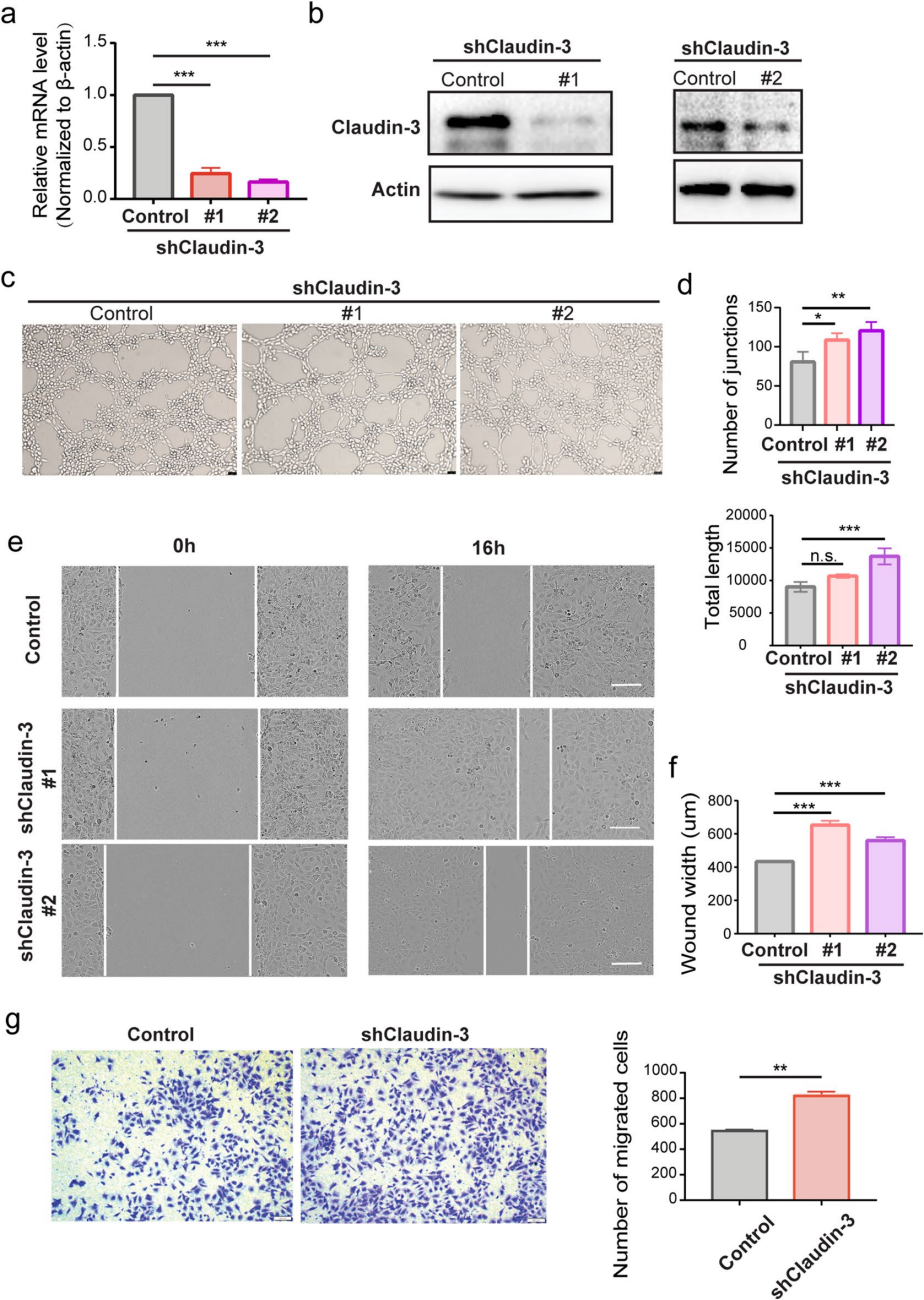
Knockdown of claudin-3 in SVEC4-10 cells promotes cell migration. (**a**) SVEC4-10 cells were transfected with two independent shRNA plasmids to knockdown claudin-3 protein (shClaudin-3-1 & shClaudin-3-2) accompanied with the transfection of control plasmid (Control). RT-PCR analysis was used to examine the mRNA expression in the two knockdown cells compared to control cells. (***p < 0.001) (n = 3 per group) (**b**) Western blot analysis indicated the knockdown of claudin-3 protein in SVEC4-10 cell. (**c**) Representative images of tube formation of SVEC4-10 cells transfected with shClaudin-3-1, shClaudin-3-2, and control. Scale bar, 100 μm. (**d**) Number of junctions and total length were analyzed as indicators of tube formation ability via the ImageJ. (n.s. no significance, ***p < 0.001) (n = 3 per group) (**e**) Scratching assay was conducted and the photos were taken at 0 h and 16 h under a microscope. Scale bar, 100 μm. (**f**) Wound width was quantified and analyzed. (***p < 0.01) (n = 5 per group) (**g**) Transwell assay was conducted and the representative images were taken. Scale bar, 100 μm. Cells on the bottom side were counted and analyzed by t-test. (**p < 0.01) (n = 5 per group).

We detected the expression of claudin-3 on LEC line SVEC4-10 and constructed claudin-3 knockdown in SVEC4-10 cells by transfecting two independent shRNAs for in vitro experiments (Fig. 3a,b). Since in vitro assay of tube formation is commonly used as a model of angiogenesis or lymphangiogenesis to monitor endothelial cells assembly^26^. We tested tube formation in our constructed cells and found that knockdown of claudin-3 in SVEC4-10 cells increased tube formation (Fig. 3c,d). It is known that LECs survival, proliferation and migration contribute to lymphangiogenesis^30,31^. Wound healing assay showed that knockdown of claudin-3 in SVEC4-10 cells led to increased cell migration (Fig. 3e,f), which was further confirmed by the transwell assay with consistent result (Fig. 3g). We also used CCK8 assay and found that knockdown of claudin-3 in SVEC4-10 cells increased cell proliferation (Supplementary Fig. 4a). These results suggest that knockdown of claudin-3 could promote lymphangiogenesis in vitro.

**Figure 4:**
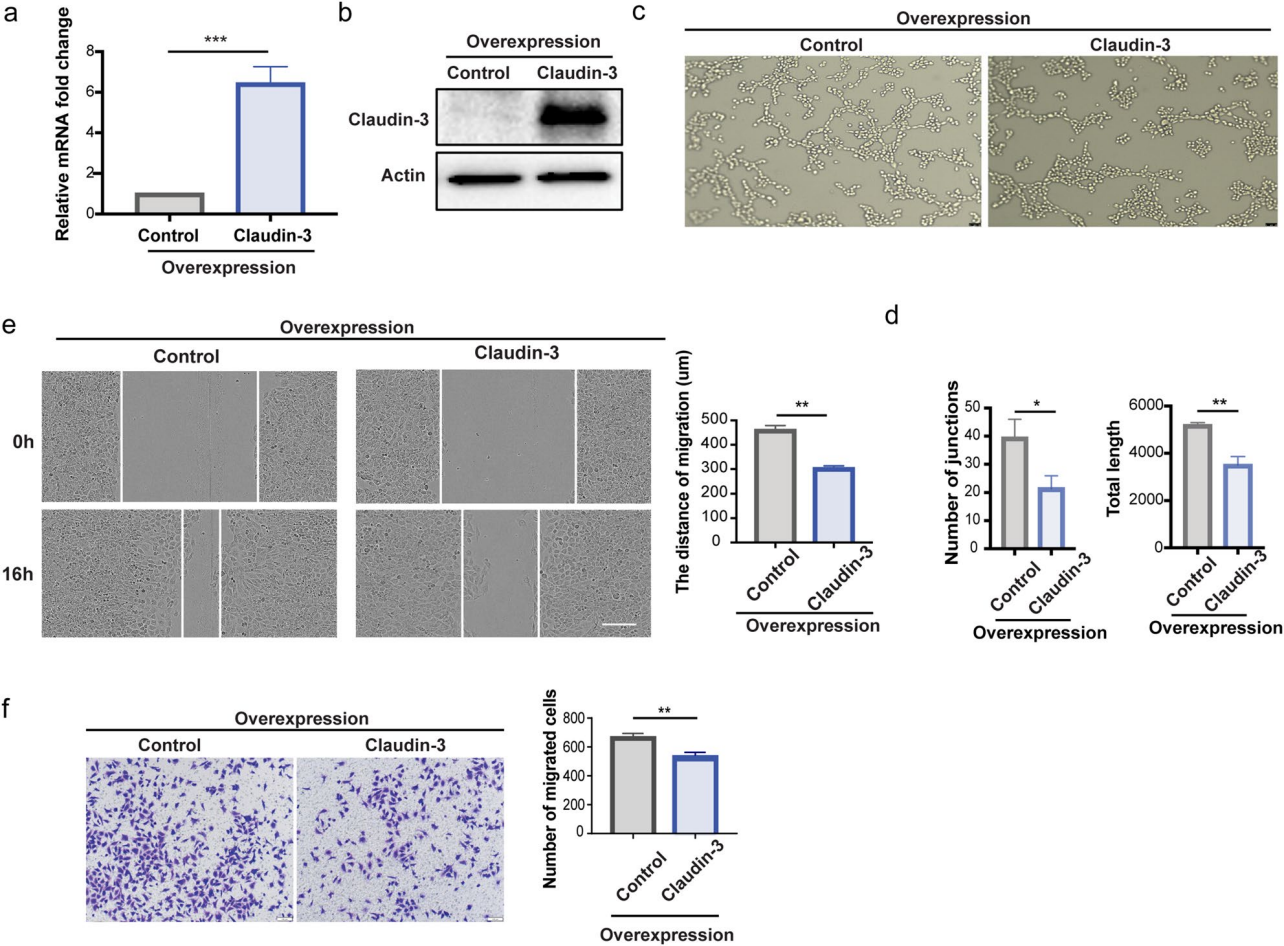
Overexpression of claudin-3 in SVEC4-10 cells inhibits cell migration. (**a**) SVEC4-10 cells were transfected with claudin-3 plasmid to overexpress the protein accompanied with the transfection of control plasmid. RT-PCR analysis was used to examine the mRNA expression in the overexpression and control cells. (***p < 0.001) (n = 3 per group) (**b**) Western blot analysis indicated the overexpression of claudin-3 protein in SVEC4-10 cell. (**c**) Representative images of tube formation of SVEC4-10 cells transfected with control and claudin-3 overexpression plasmids. Scale bar, 100 μm. (**d**) Number of junctions and total length were analyzed as indicators of tube formation ability via the ImageJ. (*p < 0.05, **p < 0.01) (n = 3 per group) (**e**) Scratching assay was conducted and the photos were taken under a microscope. Scale bar, 100 μm. Wound width was quantified and analyzed by t-test. (**p < 0.01) (n = 5 per group) (**f**) Transwell assay was conducted and the representative images were taken. Scale bar, 100 μm. Cells on the bottom side were counted and analyzed by t-test. (**p < 0.01) (n = 5 per group).

To confirm the functions of claudin-3 in SVEC4-10 cells, we established the overexpression of claudin-3 in SVEC4-10 cells (Fig. 4a,b). The overexpression of claudin-3 in SVEC4-10 cells ablated tube formation (Fig. 4c,d). Wound healing assay and transwell assay showed that overexpression of claudin-3 in SVEC4-10 cells suppressed cell migration (Fig. 4e,f). CCK8 assay also showed that the overexpression of claudin-3 in SVEC4-10 cells slightly inhibited cell proliferation ((Supplementary Fig. 4b). These results suggest that overexpression of claudin-3 could inhibit SVEC4-10 cell tube formation by suppressing cell migration.

### Claudin-3 inhibits lymphatic endothelial cell migration by regulating the PI3K signaling pathway

**Figure 5:**
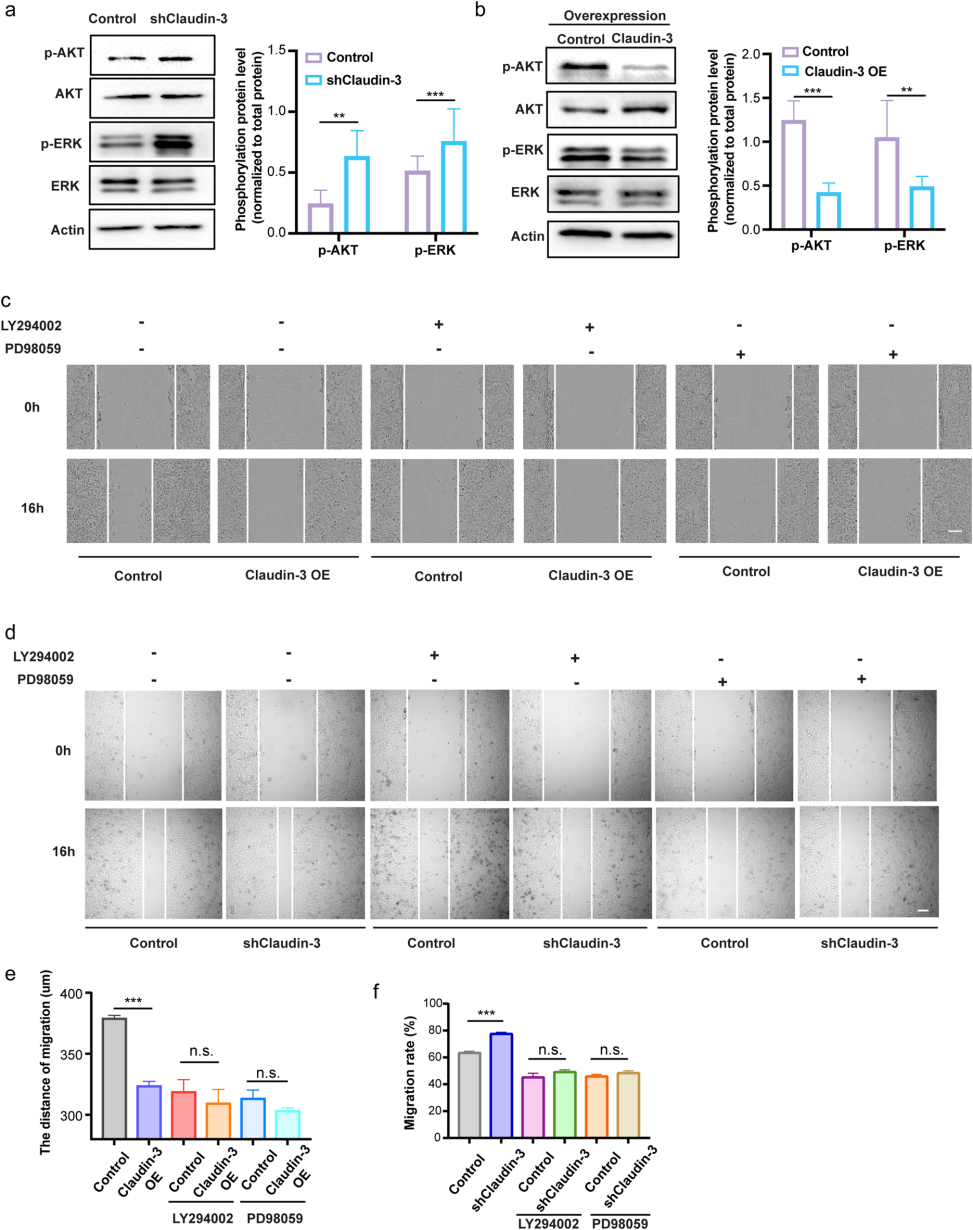
Knockdown of claudin-3 in SVEC4-10 cell activated the PI3K signaling pathway. (**a**, **b**) Western blot analysis was conducted to detect the phosphorylation of AKT and ERK in transfected SVEC4-10 cells as indicated. Total AKT and ERK were detected for normalization. (**p < 0.01, ***p < 0.001) (n = 3 per group) (**c**) SVEC4-10 Control and OE cells were pretreated with the inhibitor of PI3K (LY294002) (10 μM) or ERK (PD98059) (10 μM). Scratching assay was conducted and the photos were taken under a microscope at the indicated time. Scale bar, 100 µm. (**d**) SVEC4-10 Control and knockdown cells (shClaudin-3) were pretreated with the inhibitor of PI3K (LY294002) (10 μM) or ERK (PD98059) (10 μM). Scratching assay was conducted and the photos were taken under a microscope at the indicated time. Scale bar, 500 µm. (**e**) Wound width of result in (c) was quantified and analyzed. (n.s.: no significance; ***p < 0.01) (n = 5 per group) (**f**) Wound width of result in (**d**) was quantified and analyzed. (n.s.: no significance; ***p < 0.01) (n = 5 per group).

Since the PI3K signaling pathway is reported to regulate lymphangiogenesis in many tumors^32,33^, we then checked the phosphorylation of AKT and ERK in SVEC4-10 cells with differentially expressed claudin-3. Our result showed that the knockdown of claudin-3 increased the phosphorylation of AKT and ERK while overexpression of claudin-3 exerted an opposite effect (Fig. 5a,b). To confirm whether activation of the PI3K pathway was related to the effect of claudin-3 on LECs, we applied an inhibitor of PI3K (LY294002) or an inhibitor of ERK (PD98059). The wound healing assay showed that both the two inhibitors suppressed claudin-3 mediated SVEC4-10 cells migration (Fig. 5c–f). Taken together, the results show that claudin-3 inhibits SVEC4-10 cells migration by inhibiting the PI3K signaling pathway.

### Claudin-3 expression on SVEC4-10 cells is regulated by VEGF-C in the tumor microenvironment

**Figure 6:**
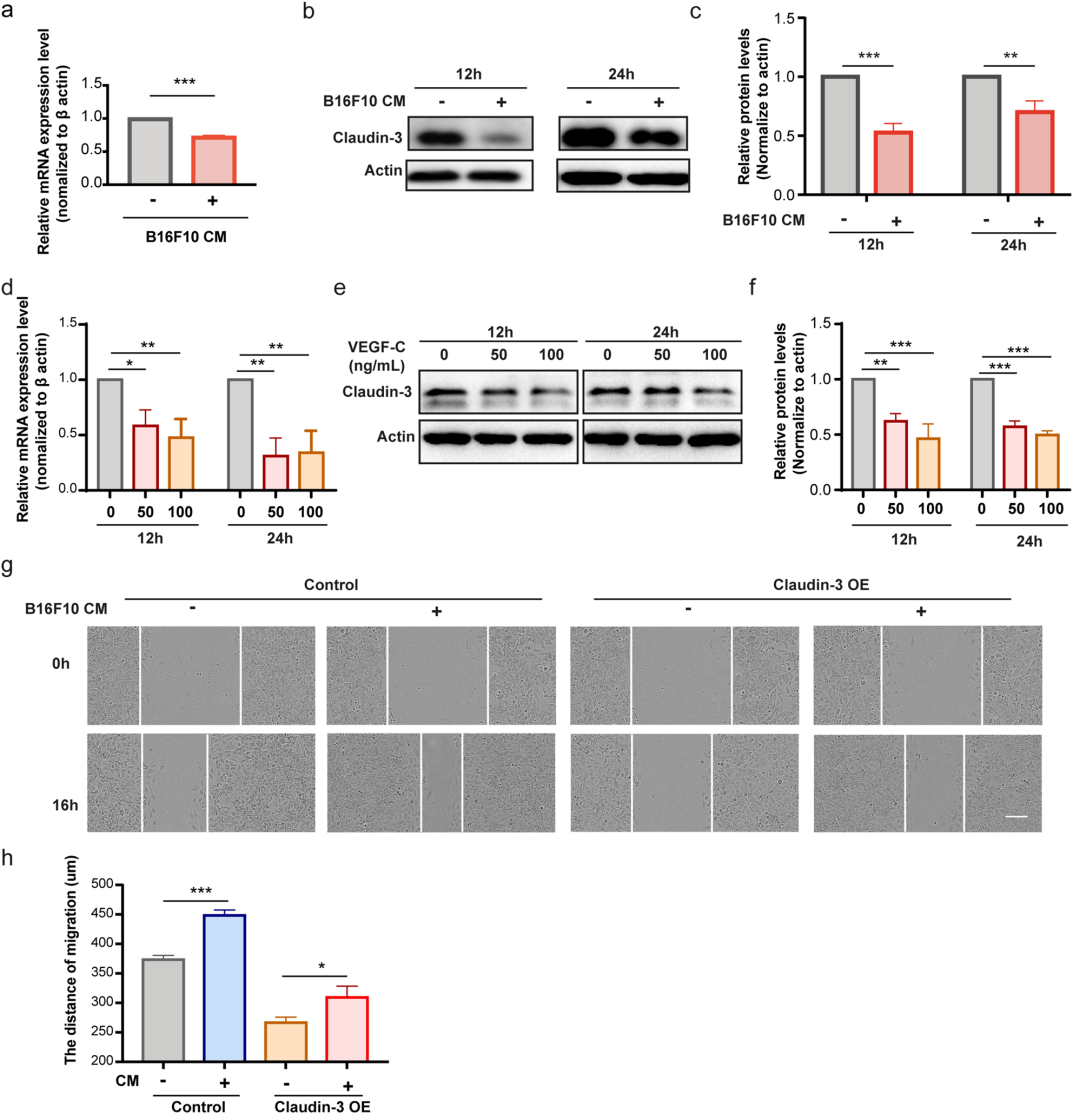
VEGF-C is sufficient to downregulate claudin-3 expression on SVEC4-10 cells. (**a**) SVEC4-10 cells were co-cultured with conditional medium (CM) from B16F10 cells for the indicated time. RT-PCR analysis was used to examine the mRNA expression of claudin-3. (***p < 0.001) (n = 3 per group) (**b**) Western blot analysis indicated the protein expression level of claudin-3 protein in SVEC4-10 cell. (**c**) Quantitative analysis for western blot results. (**p < 0.01, ***p < 0.001) (n = 3 per group) (**d**) SVEC4-10 cells were treated with 0 ng/mL, 50 ng/mL or 100 ng/mL VEGF-C for 12 h or 24 h. RT-PCR analysis was used to examine the mRNA expression of claudin-3. (*p < 0.05, **p < 0.01) (n = 3 per group) (**e**) Western blot analysis indicated the protein expression level of claudin-3 protein in SVEC4-10 cell. (**f**) Quantitative analysis for western blot results. (**p < 0.01, ***p < 0.001) (n = 3 per group) (**g**) Wound healing assay of SVEC4-10 cultured with CM from the B16F10 cells and normal control. The ratio of CM to fresh complete medium was 1:3. Photos were taken at the beginning and 16 h later. Scale bar, 100 µm. (**h**) Wound width was quantified and analyzed by t-test. (*p < 0.05, ***p < 0.001) (n = 5 per group).

We were wondering whether tumor microenvironment contributed to the alteration of claudin-3 expression. So B16F10 culture medium was collected and added to the SVEC4-10 cell culture. It was found that the conditional medium downregulated the expression of claudin-3 (Fig. 6a–c). It is known that VEGF-C/VEGFR3 axis is perhaps the most central pathway for lymphangiogenesis^34,35^. In addition, previous studies have found that VEGF-C is overexpressed in B16F10 cells^36,37^. We then found that VEGF-C treatment on SVEC4-10 cells could downregulate the expression level of claudin-3 (Fig. 6d–f). We also detected the migration effect of claudin-3 on LECs by applying the conditional medium. The wound healing assay showed that the expression of claudin-3 was required for SVEC4-10 cells migration, which could be affected by B16F10 conditional medium (Fig. 6g,h). The above results showed that VEGF-C in the tumor microenvironment could downregulated the expression of claudin-3, which further affected the LECs migratory ability.

## Discussion

**Figure 7:**
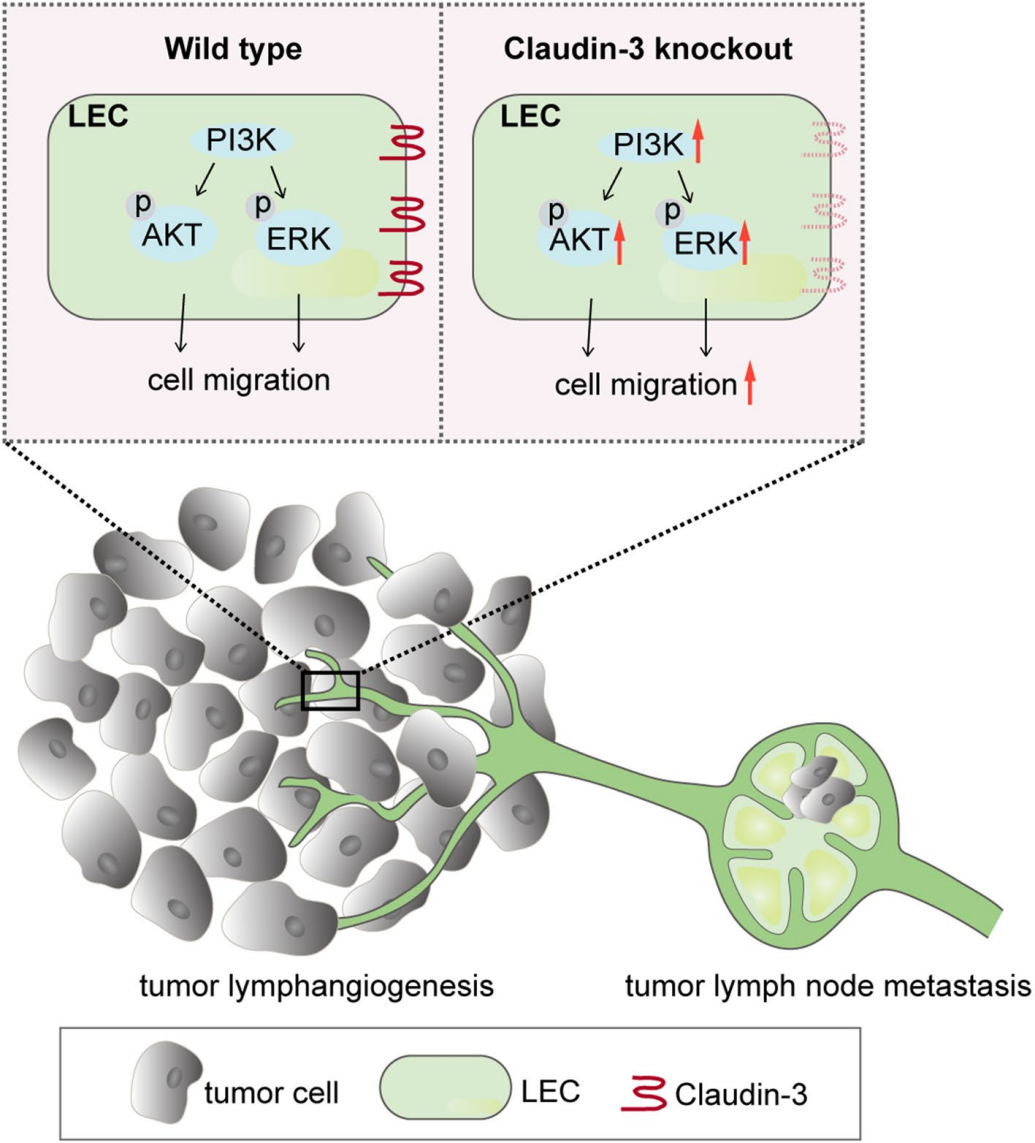
Schematic illustration of claudin-3 mediated melanoma tumor lymphangiogenesis. Claudin-3 on LEC affects cell migration via the PI3K signaling pathway. Loss of claudin-3 leads to increased phosphorylation of AKT and ERK leading to increased tumor lymphangiogenesis accompanied with more melanoma cell B16F10 metastasis into draining lymph nodes. Created with Adobe Illustrator (CC 2017; https://www.adobe.com/products/illustrator.html).

Claudin-3 is an important TJ protein on human epithelial and endothelial cell membranes, which can control the bypass transport of water, macromolecular substances and immune cells across barriers^17^. Alterations in the expression of claudin-3 protein have been reported in several tumors, and a relationship to tumor metastasis has been found^21^. The expression of claudin-3 in ovarian, prostate, breast, pancreatic and lung cancers could promote tumor invasion and development, but the effect of claudin-3 on melanoma progression and metastasis has not been addressed so far. In this study, we used claudin-3^−/−^ mice to establish a popliteal lymph node metastasis model of B16F10 melanoma. Our results revealed that loss of claudin-3 increased the metastasis of B16F10 melanoma cells into the draining lymph nodes in comparison with claudin-3^+/+^ control mice. Furthermore, we observed an increased number of lymphatic vessels in B16F10 transplantation tumor of claudin-3^/−^ mice while the density of blood vessels remained unchanged. In vitro study found that claudin-3 expression regulated SVEC4-10 cell activities associated with lymphangiogenesis (Fig. 7). Our results suggest that the expression of claudin-3 protein in the tumor microenvironment is closely related to lymphangiogenesis by affecting LECs activity.

Previous studies have shown that the expression of claudin-3 on epithelial cells contributes to the tumor progression by affecting certain signaling pathways. However, general functions of claudin-3 on endothelial cells, especially LECs are yet to be defined. It was reported that claudin-3 and other tight junction proteins found in the endothelium^38^ seal the paracellular cleft between the cells by homophilic and heterophilic protein binding^39^. Consequently, the tumor cells cannot be released from the lymphatic system into the parenchyma. We are interested to explore the expression and function of claudin-3 on LECs that contribute to melanoma lymphangiogenesis. Based on our results, both the tumor conditional medium and VEGF-C are sufficient to downregulating the expression of claudin-3 on LEC, which further suppresses tumor lymphangiogenesis. Our results indicated that the tumor microenvironment could affect lymphangiogenesis in part through the downregulation of claudin-3. We also explore that claudin-3 is involved in the regulation of LECs migraion via the PI3K signaling pathway. The PI3K signaling pathway has been widely studied in tumor lymphangiogenesis^9,32^. The exquisite molecular mechanisms on how claudin-3 functions via the PI3K signaling pathway will be further investigated.

It has been found that the absence of claudin-3 could influence expression levels of other tight junction molecules in vivo*.* For example, Castrodias et al. observed enhanced expression levels of claudin-1 protein in choroid plexus samples from claudin-3^−/−^ C57BL/6J mice when compared to that from wildtype mice^26^. Nevertheless, in one study about loss of claudin-3 expression in colon cancer, authors found that claudin-3 depletion did not affect colonic expression or cellular localization of cluadin-1, -2 or -7 proteins, known to be altered in colon cancer^23^. In another work about claudin-3 in multiple sclerosis, authors also found a normal tight and adherence junction expression profile like beta-catenin, claudin-11 and ZO-1 at the choroid plexus in claudin-3 null mice^27^. The compensational effects of other junction proteins upon claudin-3 deficiency in our tumor model need further study. Still there is a clear LEC intrinsic effect of claudin-3 loss in our study.

This study presents evidence that loss of claudin-3 protein promotes lymphatic metastasis of B16F10 melanoma cells into draining lymph nodes accompanied with increased lymphangiogenesis. In addition, claudin-3 protein plays an important role in regulating LECs activities related to lymphangiogenesis. A more detailed elucidation of the mechanisms will clarify if this protein acts as an indicator or regulator in melanoma progression. In conclusion, this study suggests the involvement of claudin-3 processes for melanoma metastasis, and a possible mechanism is proposed. Further studies may reveal if claudin-3 is a potential target for improving the management of tumor progression and metastasis.

## Supplementary Information

Supplementary Information.

## Data Availability

All data in this study are available within this article, or the supplementary material online, or from the corresponding author upon reasonable request.

## Abbreviations

LEC: Lymphatic endothelial cell
VEGF-C: Vascular endothelial growth factor C
TJ: Tight junction
JAM-B: Junctional adhesion molecule-B
FBS: Fetal bovine serum
LN: Lymph node
IHC: Immunohistochemistry
