# Supplementary material for: Claudin-3 inhibits tumor-induced lymphangiogenesis via regulating the PI3K signaling pathway in lymphatic endothelial cells
Source: Sci Rep. 2022 Oct 19;12:17440. doi: 10.1038/s41598-022-22156-6 (PMC9581975; doi:10.1038/s41598-022-22156-6)
Supplement: Supplementary file 1 — Supplementary Information. [file 41598_2022_22156_MOESM1_ESM.pdf]

Supplementary Material

Claudin-3 inhibits tumor-induced lymphangiogenesis via regulating the PI3K signaling pathway in lymphatic endothelial cells

Ningjing Lei[1, 2], Yanru Cheng[1], Jiajia Wan[1], Rosel Blasig[3], Anqi Li[1], Yueyue Bai[1, 2], Reiner F Haseloff[3], Ingolf E Blasig[3], Linyu Zhu[1]*, Zhihai Qin[1]*

[1] Medical Research Center, The First Affiliated Hospital of Zhengzhou University, Zhengzhou University, No. 1 Jianshe Road, Zhengzhou 450001, China

[2] School of Basic Medical Sciences, Zhengzhou University, No. 100 Kexue Road, Zhengzhou 450001, China

[3] Leibniz-Forschungsinstitut für Molekulare Pharmakologie (FMP), Robert-Rössle-Straße 10, Berlin 13125, Germany

***Correspondence to:** Zhihai Qin (zhihai@zzu.edu.cn) or Linyu Zhu (zhulinyu1zzu@hotmail.com), Medical Research Center, The First Affiliated Hospital of Zhengzhou University, Zhengzhou, Henan 450052, China. Phone: +86-371-66913632; Fax: +86-371-66913632.

Supplementary Figures

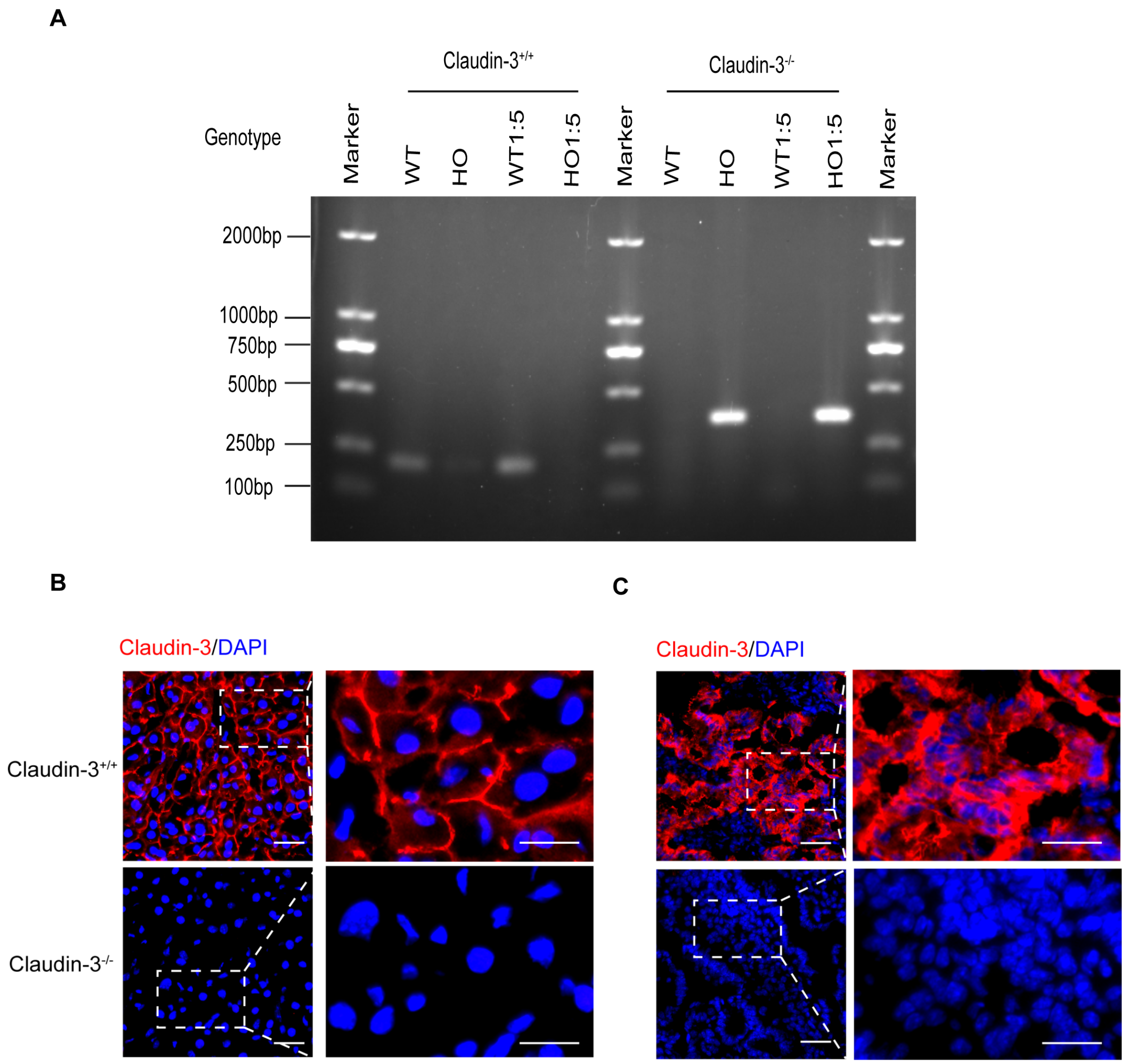

Supplementary Figure 1. Identification of homozygous claudin-3 knockout mice. A. Mice's tails were cut and digested. DNA was extracted and was analyzed by gel electrophoresis. The identified genotypes can be determined based on the expression of gene fragments. claudin-$3^{+/+}$: 225bp; claudin-$3^{-/-}$: 275bp. HO: claudin-$3^{-/-}$ homozygous; WT: claudin-$3^{+/+}$; 1:5 refer to the dilution ration of DNA as 5x for PCR analysis. B. IF staining of claudin-3 protein by anti-claudin-3 antibody (Abcam CAT#: ab15102) in liver tissues of claudin-$3^{+/+}$ and claudin-$3^{-/-}$ mice. Scale bar: 200 μm. C. IF staining of claudin-3 protein by anti-claudin-3 antibody (Abcam CAT#: ab15102) in ileum tissues of claudin-$3^{+/+}$ and claudin-$3^{-/-}$ mice. Scale bars: 200 μm.

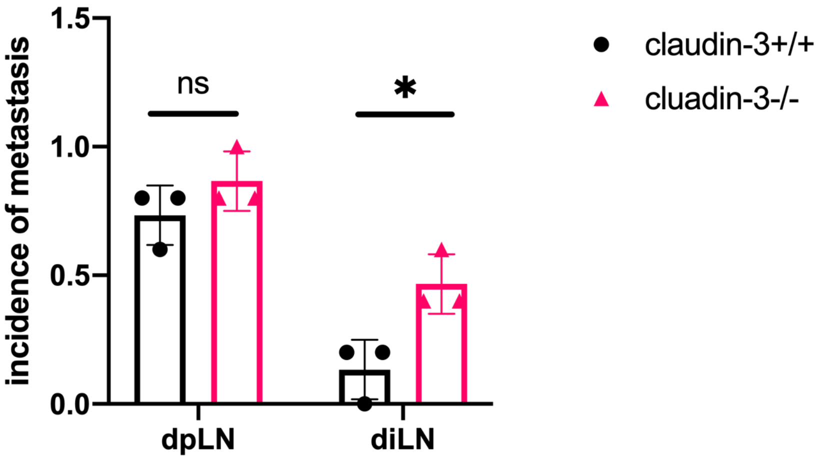

Supplementary Figure 2. Incidence of metastasized mCherry-B16F10 cells into lymph nodes. Metastasized mCherry labeled B16F10 cells into dpLNs and diLNs of claudin-3$^{-/-}$ and claudin-3$^{+/+}$ mice were analyzed on three independent experiments. dpLN: ipsilateral popliteal lymph node; diLN: ipsilateral inguinal lymph node. (ns: no significance; *$p < 0.05$) (n=5 mice per group; incidence = number of metastasized mice/5)

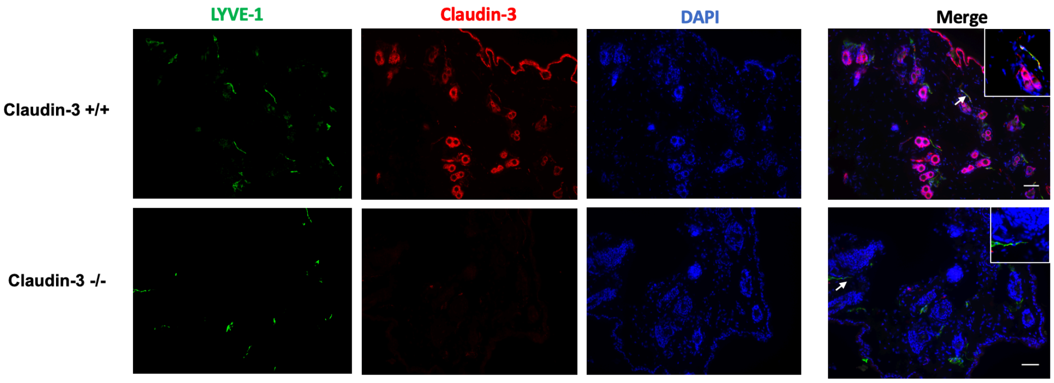

Supplementary Figure 3. Expression of claudin-3 on skin lymphatic vessels. Co-staining of skin tissue from C57BL/6 claudin3$^{+/+}$ and claudin3$^{-/-}$ mice with anti-LYVE-1 (Abcam CAT#: ab14917) and anti-claudin-3 (Abcam CAT#: ab15102). Scale bar, 500 µm.

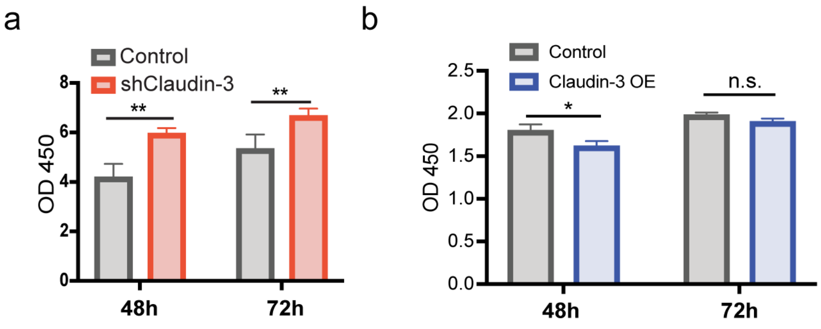

Supplementary Figure 4. Transfection of claudin-3 in SVEC4-10 cells affects cell proliferation. a. CCK-8 analysis detected the cell proliferation in SVEC4-10 control and shClaudin-3 cells at indicated time. b. CCK-8 analysis detected the cell proliferation in SVEC4-10 control and Claudin-3 overexpression cells (Claudin-3 OE) at indicated time. (ns: no significance; *p<0.05, **p<0.01) (n=5 per group)

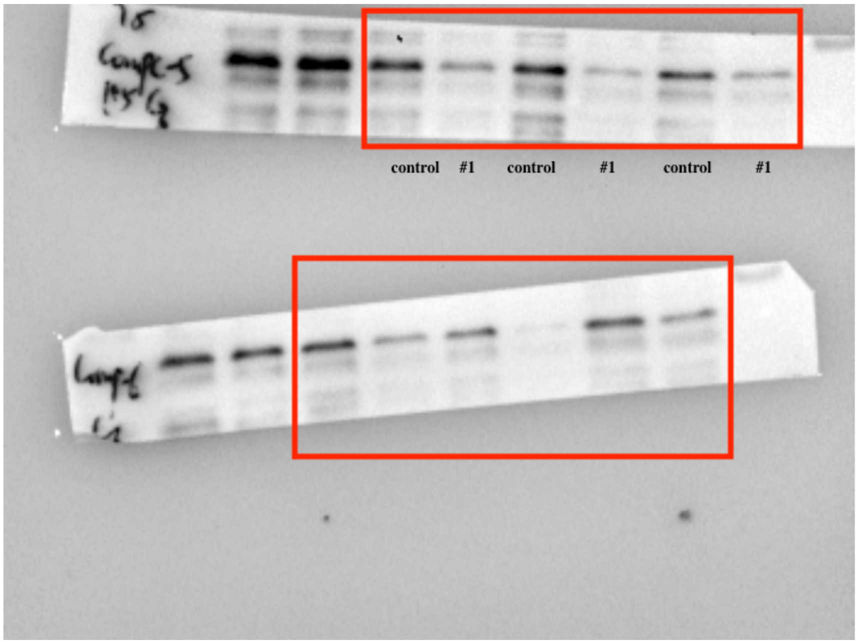

Original image of Fig 3b: claudin-3 control and knockdown; claudin-3 (20 kDa)

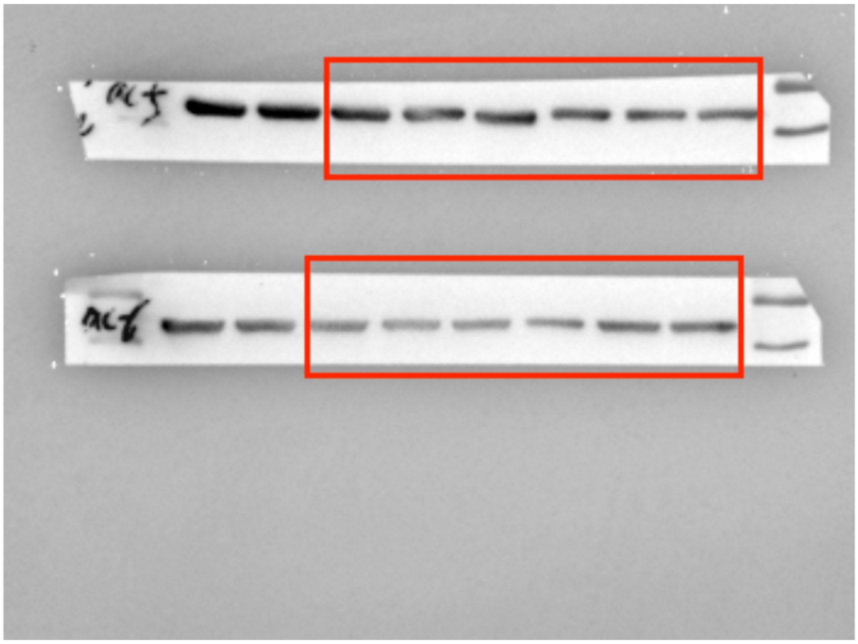

Original image of Fig 3b: actin (42 kDa)

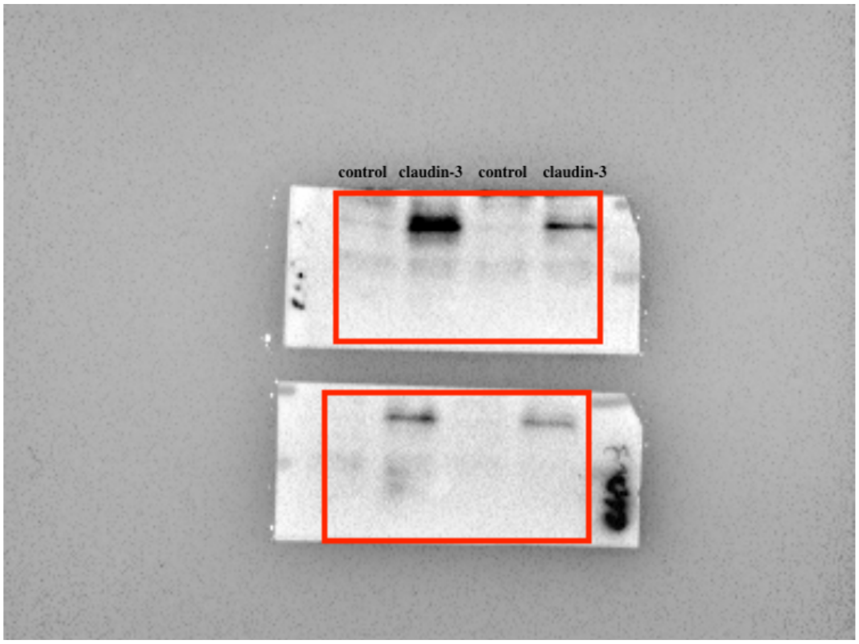

Original image of Fig 4b: claudin-3 control and overexpression; claudin-3 (20 kDa)

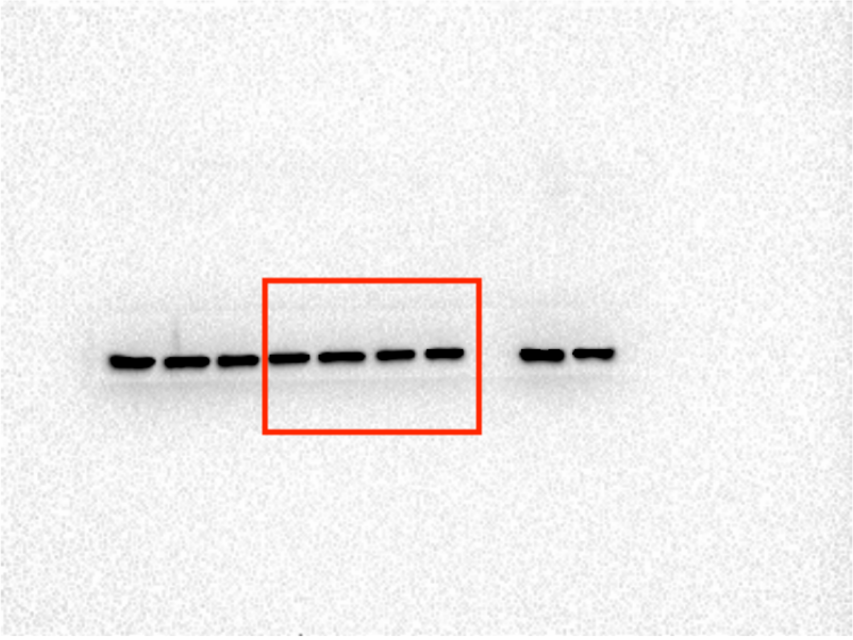

Original image of Fig 4b: actin (42 kDa)

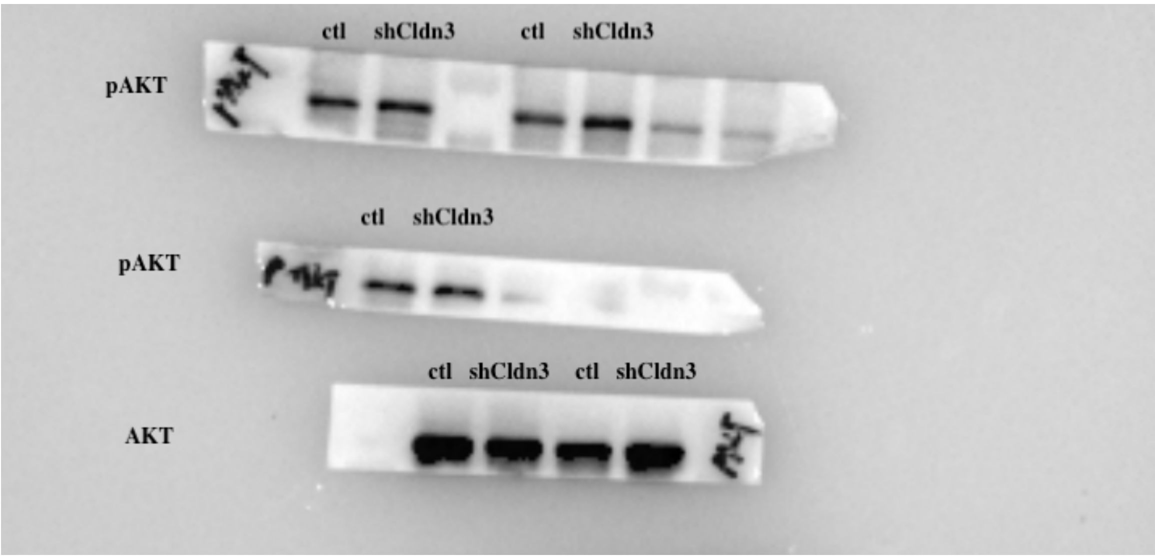

Original image of Fig 5a: phosphorylation of AKT (pAKT) and AKT (60 kDa)

SVEC4-10 control (ctl) and claudin-3 knockdown (shCldn3) cells

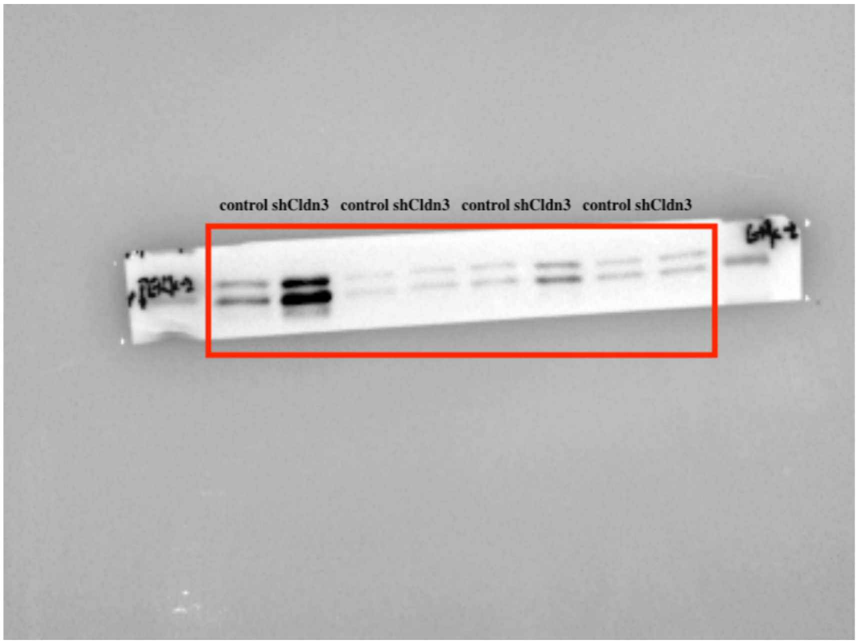

Original image of Fig 5a: phosphorylation of ERK (44, 42 kDa)

SVEC4-10 control and claudin-3 knockdown cells

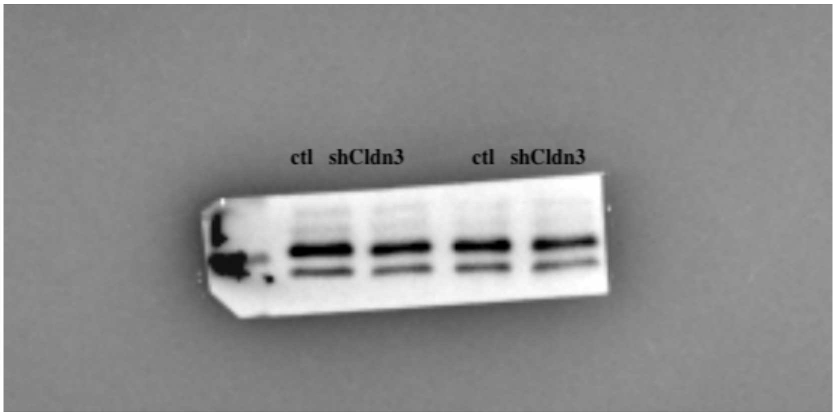

Original image of Fig 5a: total ERK protein (44, 42 kDa)

SVEC4-10 control and claudin-3 knockdown cells

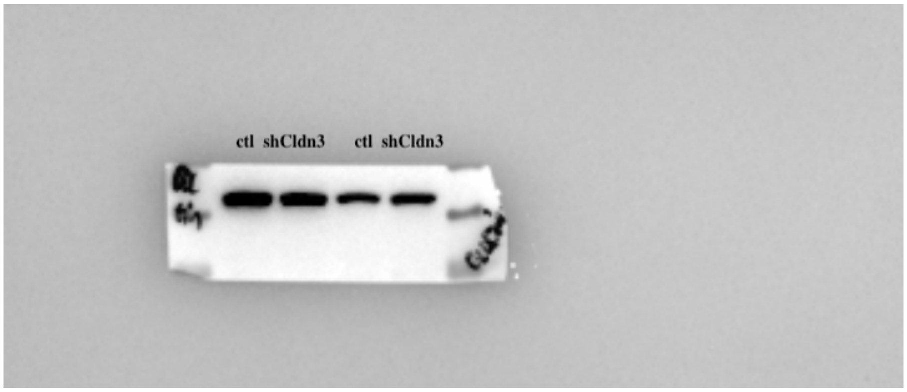

Original image of Fig 5a: actin (42 kDa)

SVEC4-10 control and claudin-3 knockdown cells

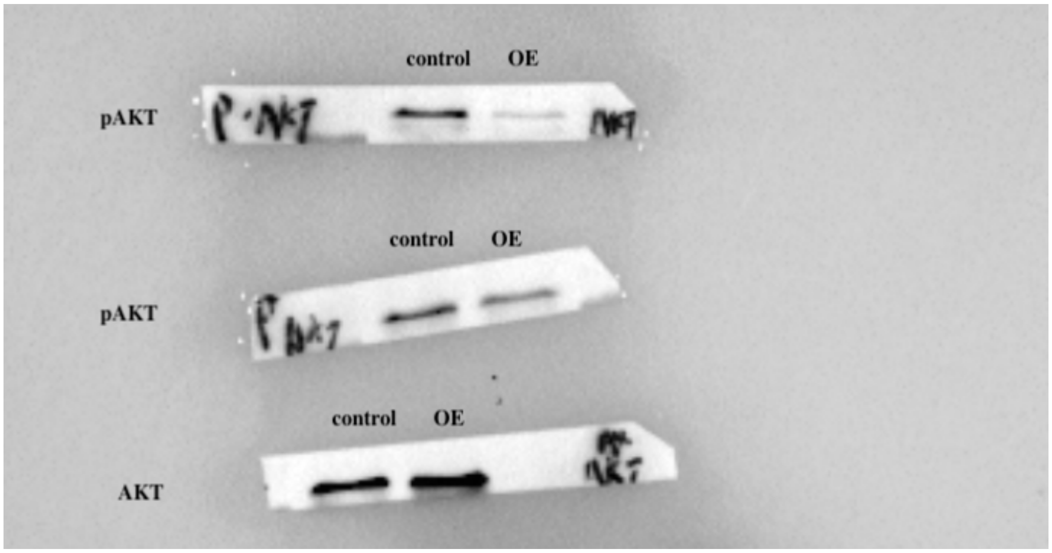

Original image of Fig 5b: phosphorylation of AKT (pAKT) and AKT (60 kDa)

SVEC4-10 control and claudin-3 overexpression (OE) cells

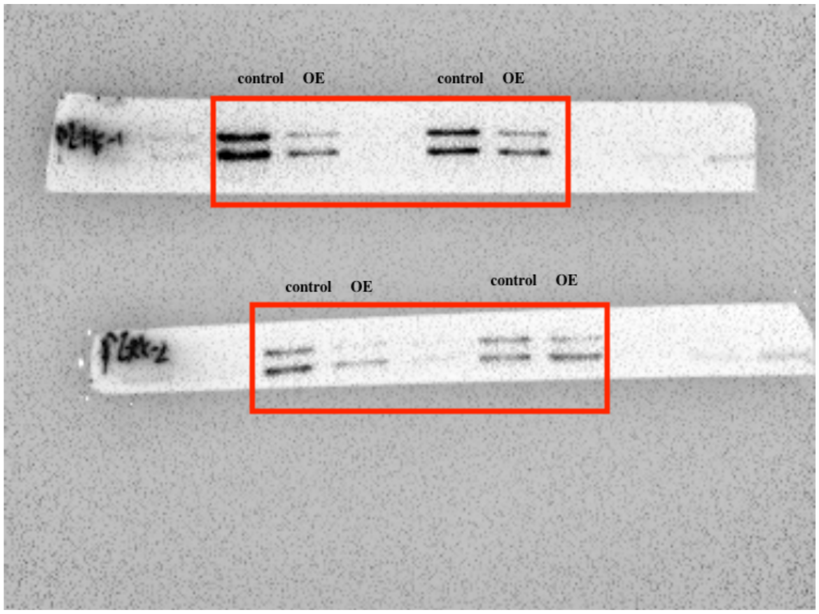

Original image of Fig 5b: phosphorylation of ERK (44, 42 kDa)

SVEC4-10 control and claudin-3 overexpression cells

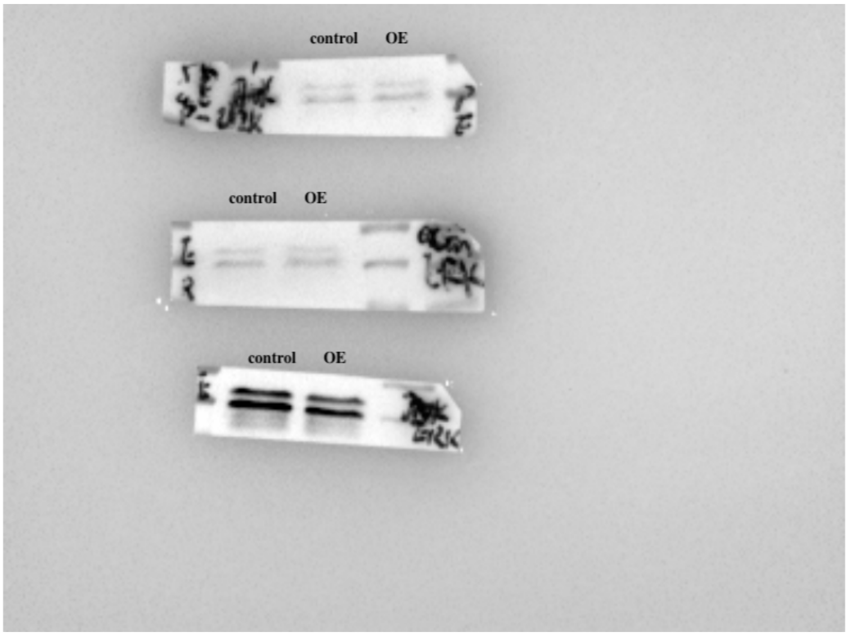

Original image of Fig 5b: total ERK protein (44, 42 kDa)

SVEC4-10 control and claudin-3 overexpression cells

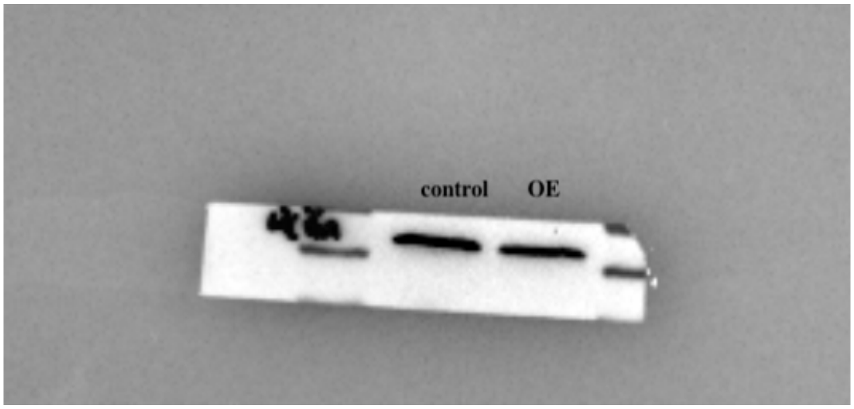

Original image of Fig 5b: actin (42 kDa)

SVEC4-10 control and claudin-3 overexpression cells

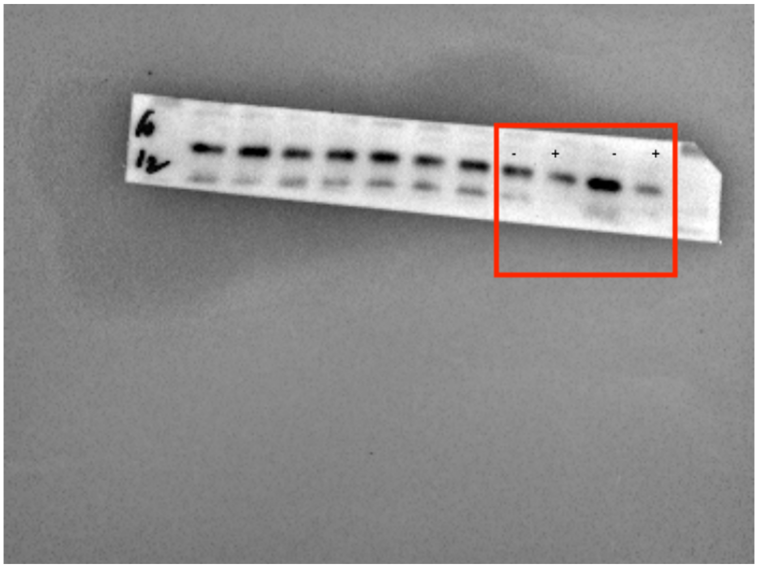

Original image of Fig 6b: claudin-3 (20 kDa)

SVEC4-10 co-cultured with conditional medium (CM) from B16F10 cells for 12h

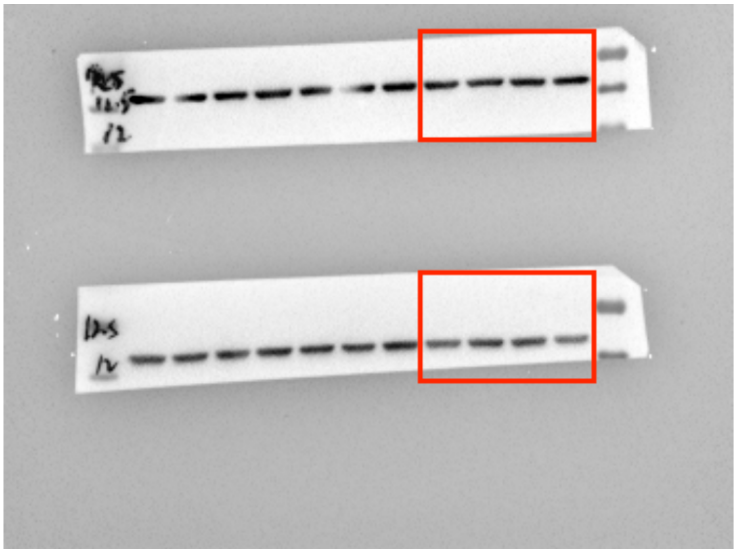

Original image of Fig 6b: actin (42 kDa)

SVEC4-10 co-cultured with conditional medium (CM) from B16F10 cells for 12h

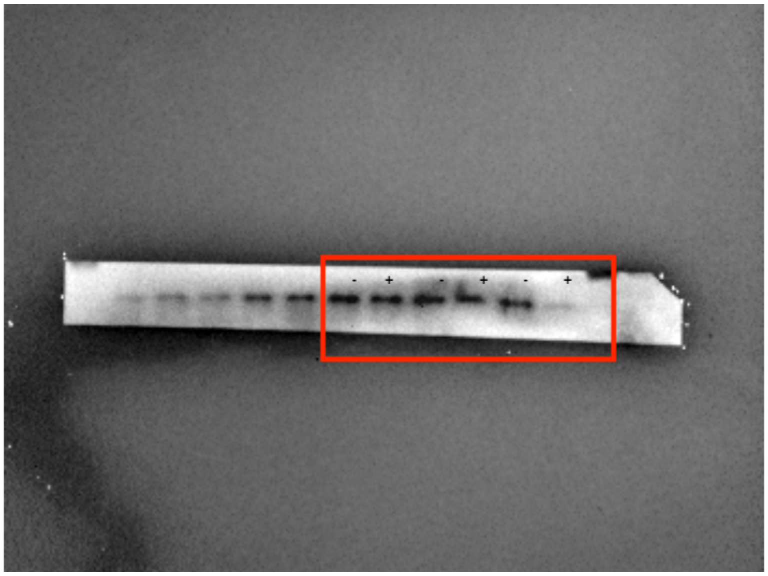

Original image of Fig 6b: claudin-3 (20 kDa)

SVEC4-10 co-cultured with conditional medium (CM) from B16F10 cells for 24h.

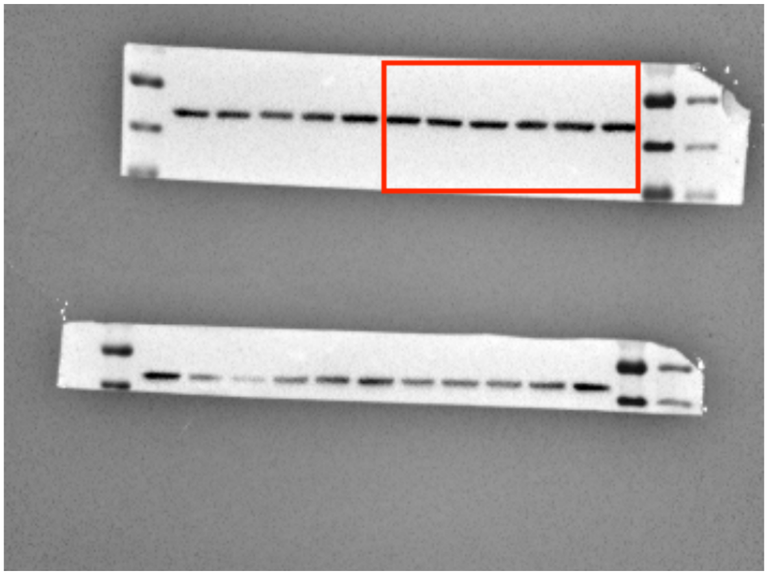

Original image of Fig 6b: actin (42 kDa)

SVEC4-10 co-cultured with conditional medium (CM) from B16F10 cells for 24h.

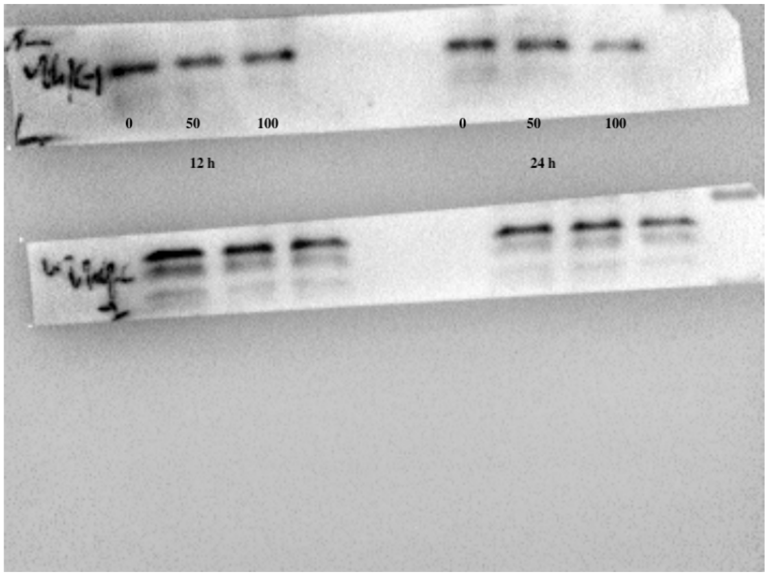

Original image of Fig 6e: claudin-3 (20 kDa)

SVEC4-10 treated with 0 ng/mL, 50ng/mL or 100 ng/mL VEGF-C for 12h or 24h.

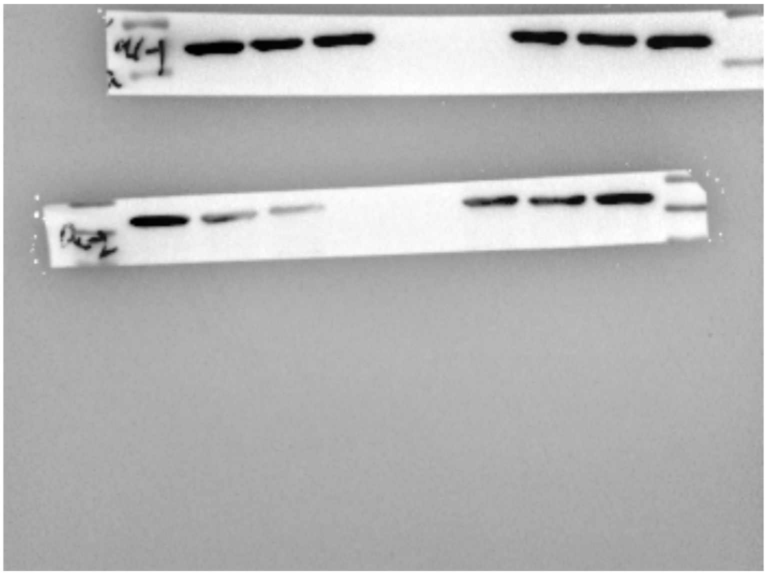

Original image of Fig 6e: actin (42 kDa)

SVEC4-10 treated with 0 ng/mL, 50ng/mL or 100 ng/mL VEGF-C for 12h or 24h.
